# Associations between Histogram Analysis Parameters Derived from DCE-MRI and Histopathological Features including Expression of EGFR, p16, VEGF, Hif1-alpha, and p53 in HNSCC

**DOI:** 10.1155/2019/5081909

**Published:** 2019-01-02

**Authors:** Hans Jonas Meyer, Leonard Leifels, Gordian Hamerla, Anne Kathrin Höhn, Alexey Surov

**Affiliations:** ^1^Department of Diagnostic and Interventional Radiology, University of Leipzig, Leipzig, Germany; ^2^Department of Pathology, University of Leipzig, Leipzig, Germany

## Abstract

**Background:**

Our purpose was to elucidate possible correlations between histogram parameters derived from dynamic contrast-enhanced MRI (DCE-MRI) with several histopathological features in head and neck squamous cell carcinomas (HNSCC).

**Methods:**

Thirty patients with primary HNSCC were prospectively acquired. Histogram analysis was derived from the DCE-MRI parameters: *K*_trans_, *K*_ep_, and *V*_e_. Additionally, in all cases, expression of human papilloma virus (p16) hypoxia-inducible factor-1-alpha (Hif1-alpha), vascular endothelial growth factor (VEGF), epidermal growth factor receptor (EGFR), and tumor suppressor protein p53 were estimated.

**Results:**

*K*_ep_ kurtosis was significantly higher in p16 tumors, and *V*_e_ min was significantly lower in p16 tumors compared to the p16 negative tumors. In the overall sample, *K*_ep_ entropy correlated well with EGFR expression (*p*=0.38, *P*=0.04). In p16 positive carcinomas, *K*_trans_ max correlated with VEGF expression (*p*=0.46, *P*=0.04), *K*_trans_ kurtosis correlated with Hif1-alpha expression (*p*=0.46, *P*=0.04), and *K*_trans_ entropy correlated with EGFR expression (*p*=0.50, *P*=0.03). Regarding *K*_ep_ parameters, mode correlated with VEGF expression (*p*=0.51, *P*=0.02), and entropy correlated with Hif1-alpha expression (*p*=0.47, *P*=0.04). In p16 negative carcinomas, *K*_ep_ mode correlated with Her2 expression (*p*=−0.72, *P*=0.03), *V*_e_ max correlated with p53 expression (*p*=−0.80, *P*=0.009), and *V*_e_ p10 correlated with EGFR expression (*p*=0.68, *P*=0.04).

**Conclusion:**

DCE-MRI can reflect several histopathological features in HNSCC. Associations between DCE-MRI and histopathology in HNSCC depend on p16 status. *K*_ep_ kurtosis and *V*_e_ min can differentiate p16 positive and p16 negative carcinomas.

## 1. Introduction

Head and neck squamous cell carcinoma (HNSCC) is a frequently occurring malignancy [1]. Previously, the role of imaging modalities was to locate the primary tumor and detect infiltration of bordering body structures and distant metastasis [2]. However, modern imaging modalities can also provide valuable information regarding tumor microstructure and might be able to predict several histopathological features in tumors [3, 4].

Dynamic contrast-enhanced MRI (DCE-MRI) is a functional imaging technique, which is able to assess tumor vascularization by measurement of sequential changes of signal intensity over time after contrast media application [5, 6]. In DCE-MRI, quantitative parameters like *K*_trans_ (volume transfer constant in min^−1^), *V*_e_ (volume fraction of the extravascular extracellular space which is dimensionless), and *K*_ep_ (rate constant in min^−1^) can be obtained [6].

Previous reports suggested that DCE-MRI can reflect tumor vessel density [6]. However, besides perfusion, DCE-MRI is also linked to cellularity, as well as to proliferation index [7, 8]. Furthermore, it has been shown that DCE-MRI can predict survival and treatment response to radiochemotherapy in HNSCC [5, 9–11]. Additionally, it can predict tumor recurrence [12] and metastatic spread [13]. Besides the prognostic information, DCE-MRI can also aid in discrimination between benign and malignant head and neck tumors [14].

Histogram analysis is used to analyze radiological images. By using this technique, every voxel of a region of interest (ROI) is issued into a histogram. Thereby, a broad spectrum of new parameters can be estimated: minimum, mean, maximum, median, mode, percentiles, kurtosis, skewness, and entropy. According to the literature, heterogeneity of the histogram might also display heterogeneity of the tumor [15].

Several histopathological parameters play an important role in HNSCC. For example, p16 expression, associated with human papilloma virus, is one of the most important prognostic factors in HNSCC [16]. Other parameters, such as vascular endothelial growth factor (VEGF), hypoxia-inducible factor-1-alpha (Hif1-alpha), epidermal growth factor receptor (EGFR), and tumor suppressor protein p53 expression, are also of prognostic relevance and might aid in treatment response prediction in HNSCC [17, 18]. Presumably, imaging might also be able to reflect these expression profiles, especially by using the more advanced histogram-based analysis. Recently, a first promising study identified statistical differences between p16 positive and p16 negative carcinomas using histogram-based parameters derived from diffusion-weighted imaging [19]. Previously, only two studies analyzed relationships between DCE-MRI and histopathological parameters like the proliferation index Ki 67 and/or tumor cellularity in HNSCC using conventional ROI-based analysis [7, 20]. Presumably, histogram-based DCE parameters may show more associations with histopathology.

Therefore, the aim of this study was to estimate whole lesion histogram parameters derived from DCE-MRI and to elucidate possible correlations with several clinically relevant histopathological features in HNSCC.

## 2. Materials and Methods

This prospective study was approved by the institutional review board (Ethics committee of the University of Leipzig, study codes 180-2007, 201-10-12072010, and 341-15-05102015). All methods were performed in accordance with the relevant guidelines and regulations. All patients gave their written informed consent.

### 2.1. Patients

For this study, 30 patients (22 men and 8 women; mean age 57.0 ± 10.6 years; range 33–77 years) with histopathological proven primary HNSCC were included into the present study. Different tumor localizations were identified: the oropharynx in 46.7% of cases, tongue in 23.3%, hypopharynx in 10%, larynx in 16.7%, and nasopharynx in 3.3% of cases. There were T3 staged cancers in 33.3% and T4 in 40% cases and only 26.7% with T1 and T2 cancers. 90% of cases were nodal positive and 10% of patients without any nodal metastases. Well and moderately differentiated tumors were identified in 36.7% of patients and poorly differentiated in 63.3%. All patients did not receive any form of cancer treatment before the investigation.

### 2.2. DCE-MRI

**Figure 1:**
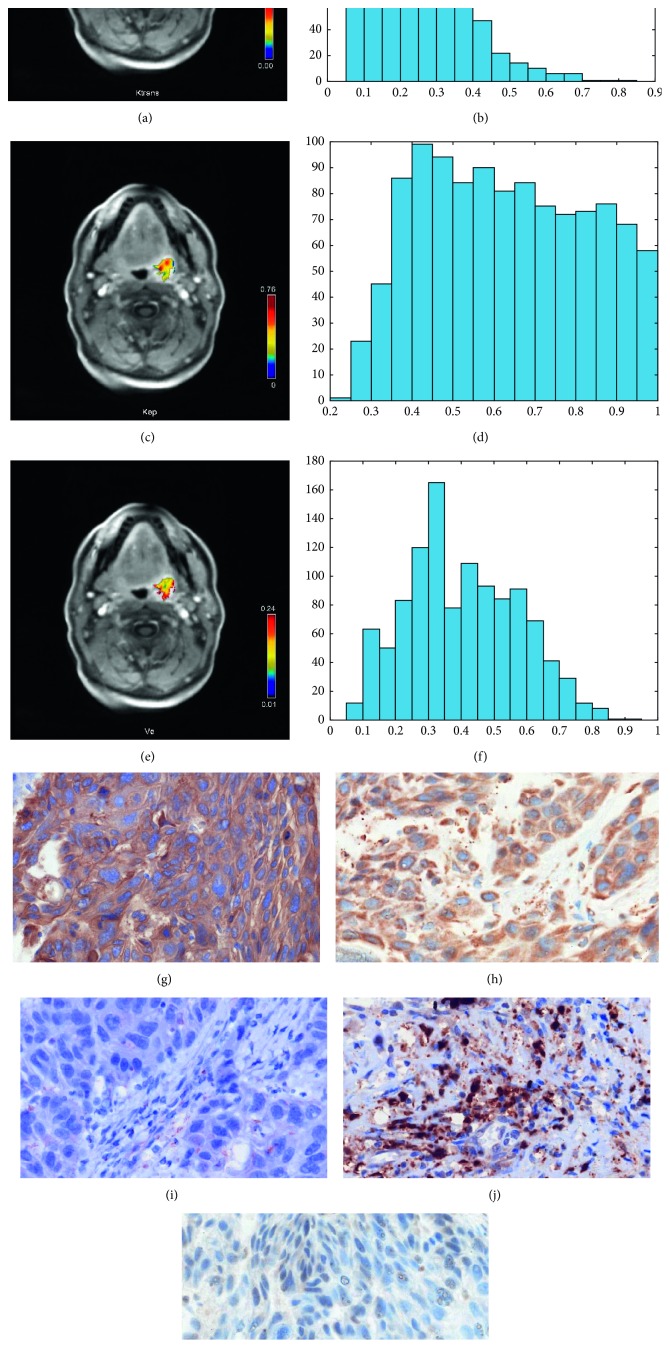
DCE-MRI and histopathological findings in a patient with histologically proven squamous cell carcinoma of the oropharynx. The p16 status is negative for this patient. (a) *K*_trans_ map of the tumor. (b) Histogram of *K*_trans_ values. The histogram analysis parameters (min^−1^) are as follows: mean = 0.25, min = 0.05, max = 0.80, p10 = 0.10, p25 = 0.16, p75 = 0.32, p90 = 0.40, median = 0.24, mode = 0.27, kurtosis = 4.5, skewness = 0.93, and entropy = 3.17. (c) *K*_ep_ map of the tumor. (d) Histogram of *K*_ep_ values. Estimated histogram analysis parameters (min^−1^) are as follows: mean = 0.63, min = 0.23, max = 1.0, p10 = 0.38, p25 = 0.46, p75 = 0.80, p90 = 0.92, median = 0.62, mode = 0.57, kurtosis = 1.89, skewness = 0.11, and entropy = 3.86. (e) *V*_e_ map of the tumor. (f) Histogram of *V*_e_ values. Estimated histogram analysis parameters are as follows: mean = 0.40, min = 0.08, max = 0.91, p10 = 0.18, p25 = 0.27, p75 = 0.53, p90 = 0.64, median = 0.39, mode = 0.25, kurtosis = 2.37, skewness = 0.29, and entropy = 3.72. (g) EGFR staining, 106866 *µ*m^2^ stained area. (h) Her2 staining, 57694 *µ*m^2^ stained area. (i) VEGF staining, 1177 *µ*m^2^ stained area. (j) Hif1-alpha staining, 27708 *µ*m^2^ stained area. (k) P53 staining, no staining is detectable in the carcinoma.

**Figure 2:**
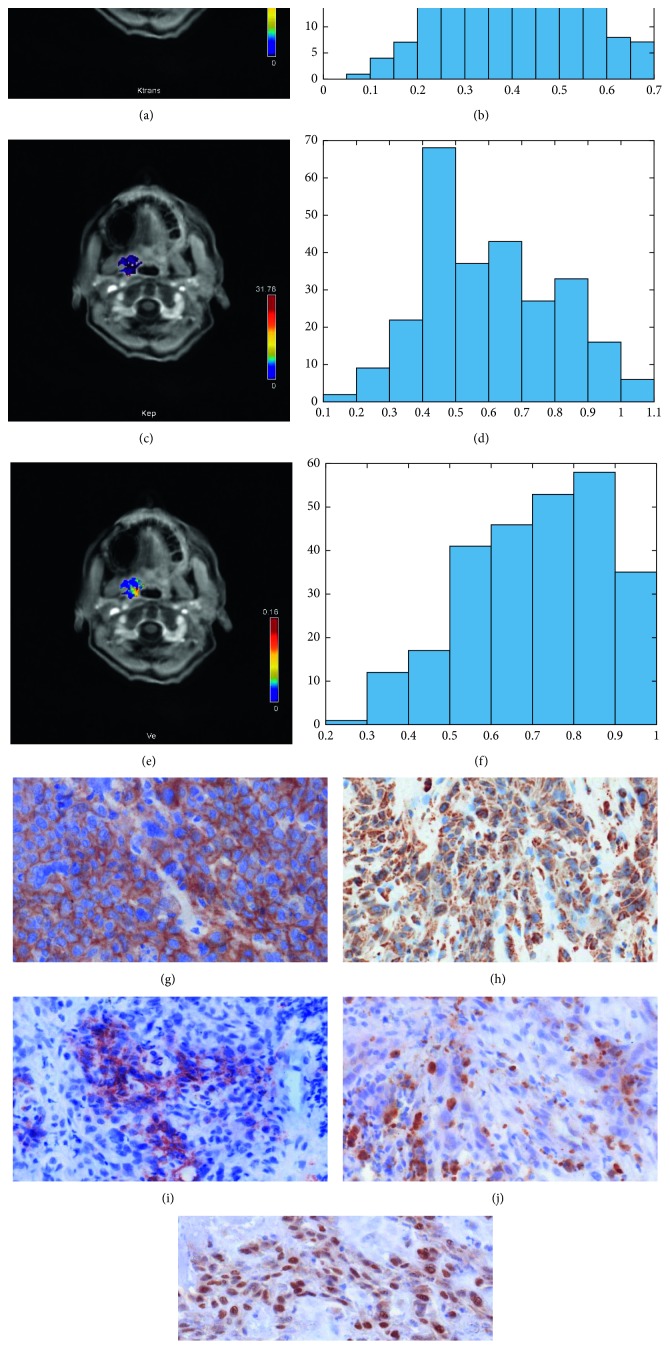
A p16 positive oropharyngeal HNSCC. (a) *K*_trans_ map of the tumor. (b) Histogram of *K*_trans_ values. The histogram analysis parameters (min^−1^) are as follows: mean = 0.42, min = 0.09, max = 0.70, p10 = 0.24, p25 = 0.35, p75 = 0.50, P90 = 0.57, median = 0.42, mode = 0.47, kurtosis = 2.78, skewness = −0.18, and entropy = 3.29. (c) *K*_ep_ map of the tumor. (d) Histogram of *K*_ep_ values. Estimated histogram analysis parameters (min^−1^) are as follows: mean = 0.60, min = 0.18, max = 1.04, p10 = 0.38, p25 = 0.45, p75 = 0.75, p90 = 0.88, median = 0.58, mode = 0.45, kurtosis = 2.20, skewness = 0.24, and entropy = 2.93. (e) *V*_e_ map of the tumor. (f) Histogram of *V*_e_ values. Estimated histogram analysis parameters are as follows: mean = 0.71, min = 0.22, max = 0.99, p10 = 0.49, p25 = 0.58, p75 = 0.86, p90 = 0.92, median = 0.73, mode = 0.63, kurtosis = 2.33, skewness = −0.38, and entropy = 2.68. (g) EGFR staining, 49020 *µ*m^2^ stained area. (h) Her2 staining, 56207 *µ*m^2^ stained area. (i) VEGF staining, 42720 *µ*m^2^ stained area. (j) Hif1-alpha staining, 11134 *µ*m^2^ stained area. (k) P53 staining, 45011 *µ*m^2^ stained area.

In all patients, dynamic contrast-enhanced (DCE) imaging was performed using T1w DCE sequences according to a imaging protocol, as reported previously (TR/TE 2.47/0.97 ms, flip angle 8°, voxel size 1.2 × 1.0 × 5.0 mm, and slice thickness 5 mm) [7, 21]. The sequence included forty scans at 6 seconds. The contrast application of 0.1 mmol gadobutrol per kg of bodyweight (Gadovist®, Bayer Healthcare, Leverkusen, Germany) started after the fifth scan with a rate of 3 ml per second (Spectris Solaris, Medrad, Bayer Healthcare, Leverkusen, Germany). The acquired images were further analyzed with Tissue 4D (Siemens Medical Systems, Erlangen, Germany), which uses a population-based technique for the arterial input function (AIF). The AIF was modelled to the gadolinium dose and according to the biexponential model of Tofts and Kermode. Finally, *K*_trans_, *V*_e_, and *K*_ep_ were calculated (for exemplary parameter images, see Figures 1 and 2).

### 2.3. Histogram Analysis

The acquired DCE-MRI data were processed with a Matlab-based application (Mathworks, Natick, MA, USA). On the *K*_trans_, *K*_ep_, and *V*_e_ maps, a volume of interest was drawn inside the tumor boundary using all slices with visible tumor areas and thus providing a whole lesion measurement. All measures were performed by one experienced author (AS, 15 years of general radiological experience). The following parameters were estimated for *K*_trans_, *K*_ep_, and *V*_e_: mean, maximum, minimum, median, mode, 10^th^, 25^th^, 75^th^, and 90^th^ percentiles, as well as kurtosis, skewness, and entropy.

### 2.4. Histopathological Findings

In every patient, the diagnosis was confirmed by tumor biopsy. The histological specimens were deparaffinized, rehydrated, and cut into 5 *μ*m slices. Moreover, the histological slices were stained by the epidermal growth factor receptor (EGFR, EMERGO Europe, clone 111.6, dilution 1 : 30), vascular endothelial growth factor (VEGF, EMERGO Europe, clone VG1, dilution 1 : 20), tumor suppressor protein p53 (DakoCytomation, Glostrup, Denmark; clone DO-7, dilution 1 : 100), hypoxia-inducible factor-1 (Hif1-alpha) (Biocare Medical, 60 Berry Dr Pacheco, CA 94553; clone EP1215Y, dilution 1 : 100), and p16 (p16 expression, CINtec Histology, Roche, Germany), as performed in our previous study [22].

Pannoramic microscope scanner (Pannoramic SCAN, 3DHISTECH Ltd., Budapest, Hungary) with Carl Zeiss objectives up to 41x bright field magnification by default was used to digitalize all specimens. In the used bottom-up technique, the whole sample was acquired at a high resolution. All slides were analyzed with Pannoramic Viewer 1.15.4 (open source software, 3D HISTECH Ltd., Budapest, Hungary), and three representative images with a magnification of ×200 were extracted from each patient.

The histopathological images were further investigated by using the ImageJ software 1.48v (National Institutes of Health Image program). The tumors were divided according to the p16 status.

Finally, expression of EGFR, VEGF, HIF1-alpha, and p53 (Figures 1 and 2) was semiautomatically estimated as a sum of stained areas (in *µ*m^2^) by using a brightness threshold.  Figure 1 displays a p16 negative, and Figure 2 shows a p16 positive carcinoma.

### 2.5. Statistical Analysis

Statistical analysis was performed using GraphPad Prism (GraphPad Software, La Jolla, CA, USA). Collected data were evaluated by means of descriptive statistics.

Spearman's correlation coefficient (*ρ*) was used to analyze associations between investigated imaging and histopathology parameters. Mann–Whitney *U* test was used for discrimination between p16 groups. *P* values below 0.05 were considered statistically significant.

## 3. Results

**Figure 3:**
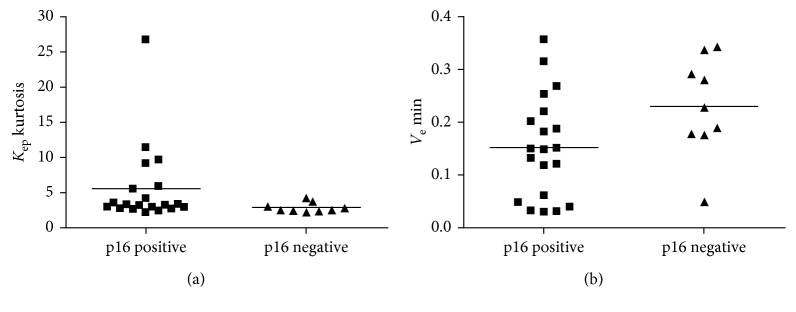
(a) Comparison between p16 and p16 negative tumors. *K*_ep_ kurtosis was significantly higher in p16 positive tumors (Mann–Whitney *U* test, *p*=0.049). (b) *V*_e_ min was significantly lower in p16 positive tumors (Mann–Whitney *U* test, *p*=0.044).

There were 10 (33.3%) p16 negative and 20 (66.7%) p16 positive tumors. *K*_ep_ kurtosis was significantly higher in p16 tumors, and *V*_e_ min was significantly lower in p16 positive tumors compared to the p16 negative tumors, *P*=0.049 and *P*=0.044, respectively (Figure 3).

**Figure 4:**
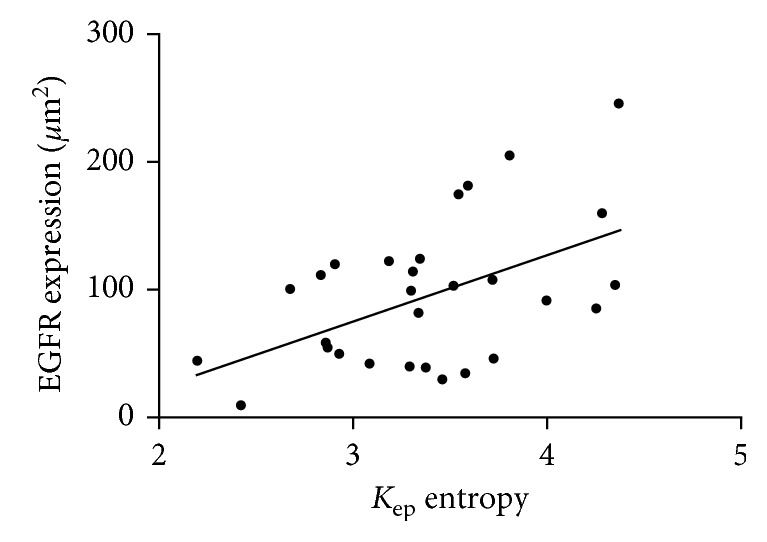
Correlation analysis between *K*_ep_ entropy and EGFR expression in the overall patient sample. Spearman's correlation coefficient (*p*=0.38, *P*=0.04).

In the overall sample, the correlation analysis revealed only one statistically significant correlation between *K*_ep_ entropy and EGFR expression (*ρ* = 0.38, *P*=0.04) (Figure 4).

In the p16 positive carcinomas, *K*_trans_ max correlated with VEGF expression (*ρ* = 0.46, *P*=0.04), *K*_trans_ kurtosis correlated with Hif1-alpha expression (*ρ* = 0.46, *P*=0.04) and *K*_trans_ entropy correlated with EGFR expression (*ρ* = 0.50, *P*=0.03). Regarding *K*_ep_ parameters, mode correlated with VEGF expression (*ρ* = 0.51, *P*=0.02), and entropy correlated with Hif1-alpha expression (*ρ* = 0.47, *P*=0.04). None of the *V*_e_ values were associated with the analyzed histochemical parameters.

In the p16 negative group, the following associations could be identified: *K*_ep_ mode correlated with Her2 expression (*ρ* = −0.72, *P*=0.03), *V*_e_ max correlated with p53 expression (*ρ* = −0.80, *P*=0.009), and *V*_e_ p10 correlated with EGFR expression (*ρ* = 0.68, *P*=0.04).

## 4. Discussion

This present study identified statistically significant associations between histogram parameters derived from DCE-MRI and different histopathological features in HNSCC. Furthermore, it showed that these relationships depended on the p16 status.

There is increasing evidence that MRI, especially using functional imaging modalities, is able to reflect tumor microstructure and to predict tumor behavior [3, 7, 8, 20]. It is widely acknowledged that DCE-MRI is associated with vascularity in tissues, especially with microvessel density as the most investigated parameter. For example, significant associations between DCE-MRI and microvessel density have been reported in experimental [23] as well as in clinical investigations [7, 24, 25].

Notably, it has been shown that different DCE parameters might also reflect different aspects of tumor microstructure [7]. So, *V*_e_ might also be strongly associated with cellularity because it reflects the amount of extracellular space, as it was exemplarily shown in a glioma model [8]. This might be one reason for the different correlations identified in the present study.

Several studies elucidated possible correlations between imaging and histopathology in HNSCC. For example, it has been shown that diffusion-weighted imaging (DWI) correlated with Ki 67 expression as well with nucleic areas [3, 26]. In another study, *K*_trans_ correlated inversely with Ki 67 expression (*r* = −0.62), whereas *V*_e_ tended to correlate with the cell count [7]. Furthermore, Jansen et al. showed that *K*_ep_ correlated statistically significant with VEGF expression (*r* = 0.808) [20].

In the present study, *K*_ep_ mode correlated with VEGF expression in p16 positive patients. Interestingly, also *K*_trans_ max correlated in a similar fashion with VEGF expression. Furthermore, *K*_trans_ max also showed a significant association with Hif1-alpha. Presumably, the maximum value of *K*_trans_ may reflect tumor areas with the highest vessel density. Therefore, the observed correlation between *K*_trans_ max and expression of VEGF is logical. Our results are in agreement with some previous reports. For example, in gliomas, also a positive correlation between VEGF and *K*_trans_ was observed [27–29].

However, some studies did not find significant associations between DCE-MRI and histopathology. For example, in breast cancer, no correlations between histogram parameters derived from DCE-MRI and VEGF expression could be identified [24].

Rasmussen et al. found associations between standardized uptake values (SUV) derived from positron emission tomography (PET) with 2-deoxy-2-[18F]fluoro-D-glucose (FDG) and histopathology in HNSCC [30]. There were negative correlations for Bcl-2 and p16 and positive with *β*-tubulin-1 index. Moreover, in another study, SUV was only associated with VEGF expression, whereas no association was found for GLUT-1, Ki 67, P53, CD68, Hif1-alpha, and CD31 [31]. Our results indicate that DCE-MRI might be more sensitive than FDG PET for prediction of histopathological features.

It is believed that the histogram-based analysis of radiological images can better reflect tumor than conventional ROI-based analysis [15]. For example, it was shown that histogram analysis of DCE and DWI can identify more correlations between parameters of these imaging modalities [32].

The present study showed that kurtosis values derived from *K*_ep_ and *V*_e_ min were significantly different in p16 positive compared to p16 negative tumors. This novel finding might be caused by several underlying tissue characteristics. In a recent study by de Perrot et al., histogram analysis derived from the ADC map was used to differentiate between p16 positive and p16 negative HNSCSS [19]. *V*_e_ is a parameter, which might be related to ADC values and cellularity [8, 30]. Interestingly, *V*_e_ min that represents voxels with the lowest extracellular space, and, presumably, areas with the highest cell density, was lower in p16 positive lesions. This finding may suggest that p16 positive tumors may show a higher cell density than p16 negative tumors. In the study by de Perrot et al., also kurtosis derived from ADC maps could distinguish p16 positive and p16 negative carcinomas [19].

These findings might be related to several causes. As reported previously, p16 positive cancers were more often nonkeratinizing and had a high Ki 67 expression [19]. Moreover, expression profiles of p16 positive and p16 negative cancers might differ significantly emphasizing their different tumor behavior. So, it was shown that expression of Eps8 is different in these subtypes of HNSCC [33]. This EGFR substrate contributes to the carcinogenesis and might be involved in invasiveness in HNSCC [31]. Interestingly, the expression of Eps8 correlated with the tumor stage and p16 status but not with anatomical localization of tumors [33]. Moreover, the expression of other histopathological parameters such as EGFR, VEGF, and NOTCH1 differ between p16 positive and negative tumors, which suggest differences in tumor angiogenesis in these entities [34]. This might be also a reason for the identified influence of p16 expression on association between imaging and histopathology.

Furthermore, it is known that p16 expression is one of the most important prognostic factors in HNSCC with a more favorable outcome for p16 positive cancers [16]. The other investigated histopathological features are also of clinical importance. So, EGFR is involved in the regulation of many cellular pathways, including cell proliferation, apoptosis, and cellular differentiation [35]. It was identified that EGFR expression is a good prognostic parameter in HNSCC [35, 36]. Furthermore, p53 regulates the activity of pathways, which lead to cell cycle arrest, senescence, or apoptosis [37]. Another parameter, namely, VEGF predicts outcome in HNSCC. VEGF overexpression has been reported as a poor indicator for patients with head and neck cancer [38]. Finally, Hif1-alpha characterizes cellular responses to hypoxic stress and is related to the neoangiogenesis [39]. Overexpression of Hif1-alpha was also significantly associated with poor survival in HNSCC [39]. Therefore, the possibility to characterize HNSCC based on imaging is very important. The identified associations between DCE-MRI parameters and several histopathological markers can be used in clinical practice.

There are several limitations of this study to address. Firstly, our patient sample size is small yet good comparable to similar studies. Secondly, we performed a whole tumor measurement for the DCE-MRI images, whereas the histopathology was investigated only on a small part of the tumor, which might limit our correlation results. Further prospective studies are needed to confirm our preliminary results.

In conclusion, the present study identified statistically significant correlations between histogram parameters derived from DCE-MRI and expression of VEGF, EGFR, p53, and Hif1-alpha in HNSCC. Associations between DCE-MRI and histopathology in HNSCC depend on the p16 status. Furthermore, *K*_ep_ kurtosis and *V*_e_ minimum can differentiate p16 positive and p16 negative carcinomas.

## Data Availability

The data used to support the findings of this study are available from the corresponding author upon request.
